# Effects of Human Synchronous Hand Movements in Eliciting a Sense of Agency and Ownership

**DOI:** 10.1038/s41598-020-59014-2

**Published:** 2020-02-06

**Authors:** Qiao Hao, Hiroki Ora, Ken-ichiro Ogawa, Shun-ichi Amano, Yoshihiro Miyake

**Affiliations:** Department of Computer Science, Tokyo Institute of Technology, Yokohama, Japan

**Keywords:** Perception, Human behaviour

## Abstract

The self is built as an entity independent from the external world using the human ability to experience the senses of agency and ownership. Humans usually experience these senses during movement. Nevertheless, researchers recently reported that another person’s synchronous mirror-symmetrical movements elicited both agency and ownership in research participants. However, it is unclear whether this elicitation was caused by the synchronicity or the mirror symmetry of the movements. To address this question, we investigated the effect of interpersonal synchronization on the self-reported sense of agency and ownership in two conditions, using movements with and without mirror symmetry. Participants performed rhythmic hand movements while viewing the experimenter’s synchronous or random hand movements, and then reported their perceptions of agency and ownership in a questionnaire. We observed that agency and ownership were significantly elicited by the experimenter’s synchronous hand movements in both conditions. The results suggested that the synchronous movements of another person—rather than mirror- or non-mirror-symmetrical movements—appear to elicit the experience of a sense of agency and ownership. The results also suggested that people could experience these senses not only from their own movements but also from another person’s synchronous movements.

## Introduction

When we move our arms in daily life, we certainly sense the execution of these movements in our bodies. People usually sense such experiences in their own movements, which provides what are known as the sense of agency and the sense of ownership^1^. These senses help us to avoid confusing our sensations with those of others (*e.g*. a cause-and-effect relationship in human behaviour) or feeling our own sensations in someone else’s body. For example, some patients suffering from asomatognosia feel that their own limbs are alien, despite tactile sensations in the ‘alien’ limb^2^. Hence, the ability to perceive self-agency and self-ownership is quite important for building the self as an entity that is independent of the external world. Surprisingly, sense of ownership was reported to be elicited in people viewing the synchronous brushing of a rubber hand while their own hands were hidden from view in the ‘rubber hand illusion’^3^. Furthermore, some researchers have found that the synchronous movements of a rubber hand, placed congruently with a participant’s hand, elicit a sense of agency and ownership in a participant^4–8^.

Interestingly, it has been reported that a sense of agency is elicited when the participant is synchronized with movements of a rubber hand rotated 180° with and without mirror symmetry, as if it was another person’s right or left hand^6–8^, whereas the sense of ownership was not elicited (Table 1). This research suggests that even though the rubber hand was in the position of another person, the participant had a sense of self-agency in relation to the rubber hand. On this topic, a recent study has indicated that people experienced both the senses of agency and ownership during interactions when viewing synchronous, mirror-symmetrical movements when seated face-to-face with an experimenter^9^, that is, another person’s movements that simultaneously included synchronous movements and mirror-symmetrical movements elicited senses of agency and ownership. However, there was no report of the effect of synchronous movements on the senses of agency and ownership in the non-mirror-symmetrical movement condition^9^. Thus, it is unclear whether synchronous movements or mirror-symmetrical movements elicited the senses of agency and ownership. Furthermore, it is suggested that synchronous movements may elicit these senses through temporal synchronous perceptions during interpersonal synchronization. That is, temporal synchronous perception could affect the multisensory integration of human perceptions, as the senses of agency^10–16^ and ownership^13,17–20^ were generated by multisensory integration. In contrast, if mirror-symmetrical movements elicit these senses, the visual information on mirrored movements during interpersonal synchronization could affect the multisensory integration of human perceptions to induce elicitation.

**Table 1:** Results of agency and ownership in previous studies.

	Interpersonal synchronization	Human–rubber hand synchronization		
	Zhou *et al*.^7^	Jenkinson & Preston^4^	Karabanov *et al*.^5^	Marotta *et al*.^6^
	R(P)–L(E); L(P)–R(E)	R(P)–L(RH)	L(P)–R(RH)	R(P)–R(RH)
Sense of agency	ο	ο	ο	ο
Sense of ownership	ο	×	×	×

R: right hand. L: left hand. P: participant. E: experimenter. RH: rubber hand.

ο: significant difference between the present and previous studies.

×: no significant difference between the present and previous studies.

To address this question, we aimed to investigate the senses of agency and ownership in relation to another person’s mirror- and non-mirror-symmetrical movements. In particular, we conducted experiments to investigate perceptions of the senses of agency and ownership when the experimenter mimicked the movements of participants’ right or left hands in combinations of four conditions: mirrored, non-mirrored, synchronous movement, and random movement.

In the mirrored condition, the experimenter used his/her opposite hand to mimic the participant’s hand movements. In the non-mirrored condition, the experimenter used the same hand to mimic the participant’s hand movements. In addition, in the synchronous-movement condition, the experimenter performed the hand movements in synchronization with those of the participant. Furthermore, in the random movement condition, the experimenter performed the hand movements in synchronization with temporally random sounds. Here, although the asynchronous condition is an important control for the synchronous condition, other studies on the rubber hand illusion and the virtual hand illusion have shown that self-agency and self-ownership can be elicited by asynchronous movements^4,21–23^. Thus, a random condition would be a better control for the synchronous condition^24^. During the tasks, participants were always asked to look at the experimenter’s open-and-close hand movements. In this study, we used a 2 × 2 × 2 experimental design, with Synchrony (synchronous vs. random), Movement type (mirrored vs. non-mirrored), and Participant’s hand (left vs. right) as the independent variables, and we tracked the time-series data on the participant’s hand movements and the experimenter’s hand movements to check whether synchronization between their hand movements had been established.

## Results

### Comparability of all the synchronous conditions

**Figure 1:**
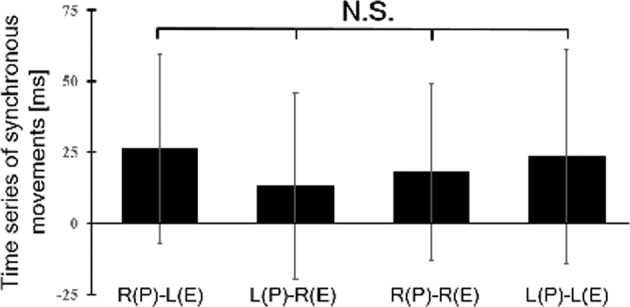
Results of time-series analysis of synchronous movements in all conditions. Error bars represent standard errors. R: right hand. L: left hand. P: the participant. E: the experimenter. N.S.: no significant difference.

To ensure the comparability of synchronous participant and experimenter movements in the mirrored and non-mirrored synchronous conditions, we monitored time-series data during the participant and experimenter movements. Then, we calculated the mean values of the intervals between participants’ and experimenters’ touches in all synchronous conditions and used a Friedman test to compare the differences between these mean values. The Friedman test indicated no differences between the synchronous conditions: χ^2^ (3, n = 32) = 4.3542, *p* = 0.23 (Fig. 1). This means that the participant and experimenter hand movements in all the mirrored and non-mirrored synchronous conditions were indeed synchronized; therefore, any differences in the agency and ownership ratings are not due to differences in hand movements in the synchronous conditions.

### Agency ratings

**Figure 2:**
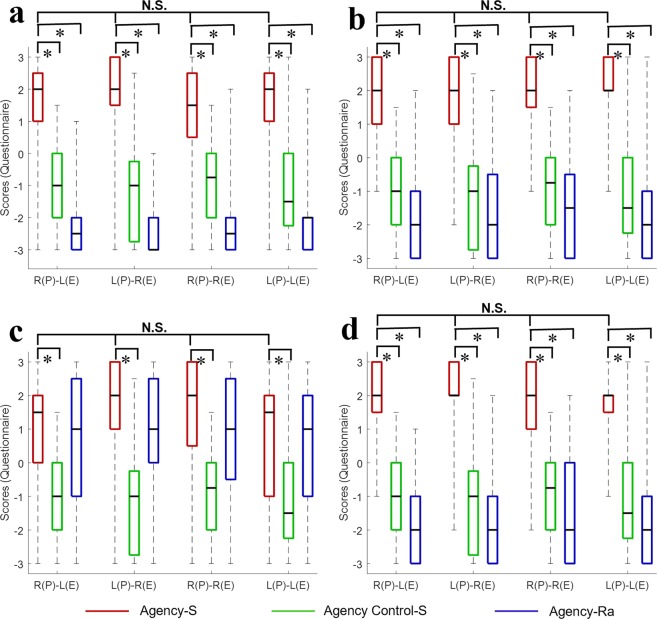
Agency results for four questions in all of the conditions, showing the results of (**a**) Q_2_, (**b**) Q_5_, (**c**) Q_8_, and (**d**) Q_11_. The four experimental conditions are shown on the abscissa, and the ordinate shows the median scores in the question on the sense of agency. Bold lines indicate the median; upper and lower limits of the box plot indicate the 75^th^ and 25^th^ percentiles, respectively. Error bars represent the entire range of the ratings of the statement. R: right hand. L: left hand. P: the participant. E: the experimenter. Agency-S: agency statement in the synchronous-movement condition. Agency Control-S: agency control statement in the synchronous-movement condition. Agency-Ra: agency statement in the random movement condition. N.S.: no significant difference. *p < 0.001.

The median and percentile (25%; 75%) ratings for perceived sense of agency are shown in Table 2 and Fig. 2. To examine the elicited sense of agency, we compared the agency statement with the corresponding control statement in synchronous conditions, and to the agency statement in the random conditions with the Wilcoxon signed-rank test (Table 3 and Fig. 2). Then, we used a Bayesian Wilcoxon signed-rank test^25^ to measure the differences in the agency statement, including Q_2_, Q_5_, Q_8_, and Q_11_, between the mirrored and non-mirrored conditions. The Bayesian analysis used the Dirichlet process prior to testing the null hypothesis. Hypothesis H_0_ is that there is no significant difference between the agency statements in the mirrored and non-mirrored conditions.

**Table 2:** Median and percentiles [25%; 75%] for agency ratings in all conditions.

Condition		Item				
		Q_2_	Q_5_	Q_8_	Q_11_	Q_C_
Synchronous condition	R(P)–L(E)	2[1;2.25]	2[1;3]	1.5[0;2]	2[1.75;3]	−1[−2;0]
	L(P)–R(E)	2[1.75;3]	2[1;3]	2[1;3]	2[2;3]	−1[−2.63;−.38]
	R(P)–R(E)	1.5[0.75;2.25]	2[1.75;3]	2[0.75;3]	2[1;3]	−1[−2;0]
	L(P)–L(E)	2[1;2.25]	2[2;3]	1.5[−1;2]	2[1.75;2]	−1.5[−2.13;0]
Random condition	R(P)–L(E)	−2.5[−3;−2]	−2[−3;−1]	1[−1;2.25]	−2[−3;−1]	−1.5[−2.63;0]
	L(P)–R(E)	−3[−3;−2]	−2[−3;−0.75]	1[0;2.25]	−2[−3;−1]	−1.5[−3;0]
	R(P)–R(E)	−2.5[−3;−2]	−1.5[−3;−0.75]	1[−0.25;2.25]	−2[−3;0]	−1[−2;1]
	L(P)–L(E)	−2[−3;−2]	−2[−3;−1]	1[−1;2]	−2[−3;−1]	−1[−2;0]

R: right hand. L: left hand. P: participant. E: experimenter. C: control statement.

**Table 3:** Comparisons between agency ratings and agency control ratings for synchronous conditions, and between agency ratings of synchronous and random conditions.

Item	Condition							
	R(P)–L(E)		L(P)–R(E)		R(P)–R(E)		L(P)–L(E)	
	Z	*P*	Z	*p*	Z	*p*	Z	*p*
Q_2_ vs. Q_C_	4.921	<0.0001	4.850	<0.0001	4.467	<0.0001	4.814	<0.0001
Q_2-S_ vs. Q_2-Ra_	4.965	<0.0001	4.982	<0.0001	4.560	<0.0001	4.892	<0.0001
Q_5_ vs. Q_C_	4.723	<0.0001	4.311	<0.0001	4.851	<0.0001	4.858	<0.0001
Q_5-S_ vs. Q_5-Ra_	4.845	<0.0001	4.865	<0.0001	4.823	<0.0001	4.912	<0.0001
Q_8_ vs. Q_C_	3.953	<0.0001	4.797	<0.0001	4.110	<0.0001	3.561	<0.001
Q_8-S_ vs. Q_8-Ra_	1.049	0.314	1.940	0.054	2.329	0.019	1.128	0.269
Q_11_ vs. Q_C_	4.629	<0.0001	4.489	<0.0001	4.449	<0.0001	4.849	<0.0001
Q_11-S_ vs. Q_11-Ra_	4.911	<0.0001	4.89	<0.0001	4.551	<0.0001	4.899	<0.0001

R: right hand. L: left hand. P: participant. E: experimenter. C: control statement. S: synchronous condition. Ra: random condition.

Q_2_^26–28^, Q_5_^8,28^, and Q_11_^26,29^ concern the sense of agency in the rubber hand illusion. Q_2_ is ‘The experimenter’s hand moved just like I wanted it to, as if it was obeying my will’. Q_5_ is ‘I felt as if I was causing the movement that I saw’. Q_11_ is ‘I felt as if I was controlling the movements of the experimenter’s hand’. The median ratings of these questions were positive in synchronous conditions (Table 2, Fig. 2a–c), indicating a subjective perception of the sense of agency. They were significantly higher than the medians of the corresponding control statements in the synchronous conditions, and the agency statements in the random conditions (all *p* < 0.0001; Table 3). Q_8_ is ‘Whenever I moved my finger, I expected the experimenter’s finger to move in the same way’^26,30,31^. The medians of Q_8_ ratings in synchronous conditions were positive (Table 2 and Fig. 2d). They were significantly higher than the corresponding control statement in the synchronous conditions (*p* < 0.001; Table 3), and higher than the agency statement in the random conditions (Table 3). Hence, the questionnaire data clearly show that participants experienced a strong sense of agency over the experimenter’s hand in all of the synchronous conditions that was not evident in the random conditions.

To compare the overall statements of agency between mirrored and non-mirrored conditions, we used the mean value of the question responses in the agency statement. Hereafter, P and E mean ‘participant’ and ‘experimenter’, respectively.

After Bayesian analysis, the Bayes factors of the P(H_0_|Data) were 0.471 (Right(P)–Left(E) vs. Right(P)–Right(E)), 0.472 (Right(P)–Left(E) vs. Left(P)–Left(E)), 0.246 (Left(P)– Right(E) vs. Right(P)–Right(E)), and 0.257 (Left(P)–Right(E) vs. Right(P)–Right(E)). According to a suggestion from Jeffreys^32^, there is anecdotal evidence of similarity between the mirrored condition of Right(P)–Left(E) and non-mirrored conditions of Right(P)–Right(E) and Left(P)–Left(E), and there is moderate evidence of similarity between the mirrored condition of Left(P)–Right(E) and non-mirrored conditions of Right(P)–Right(E) and Left(P)–Left(E). Therefore, this result supports the null hypothesis H_0,_ at least as a tendency. That is, the results showing no significant difference indicated that synchronous rather than visual information from mirrored movements elicit a sense of agency during interpersonal synchronization.

### Ownership ratings

**Figure 3:**
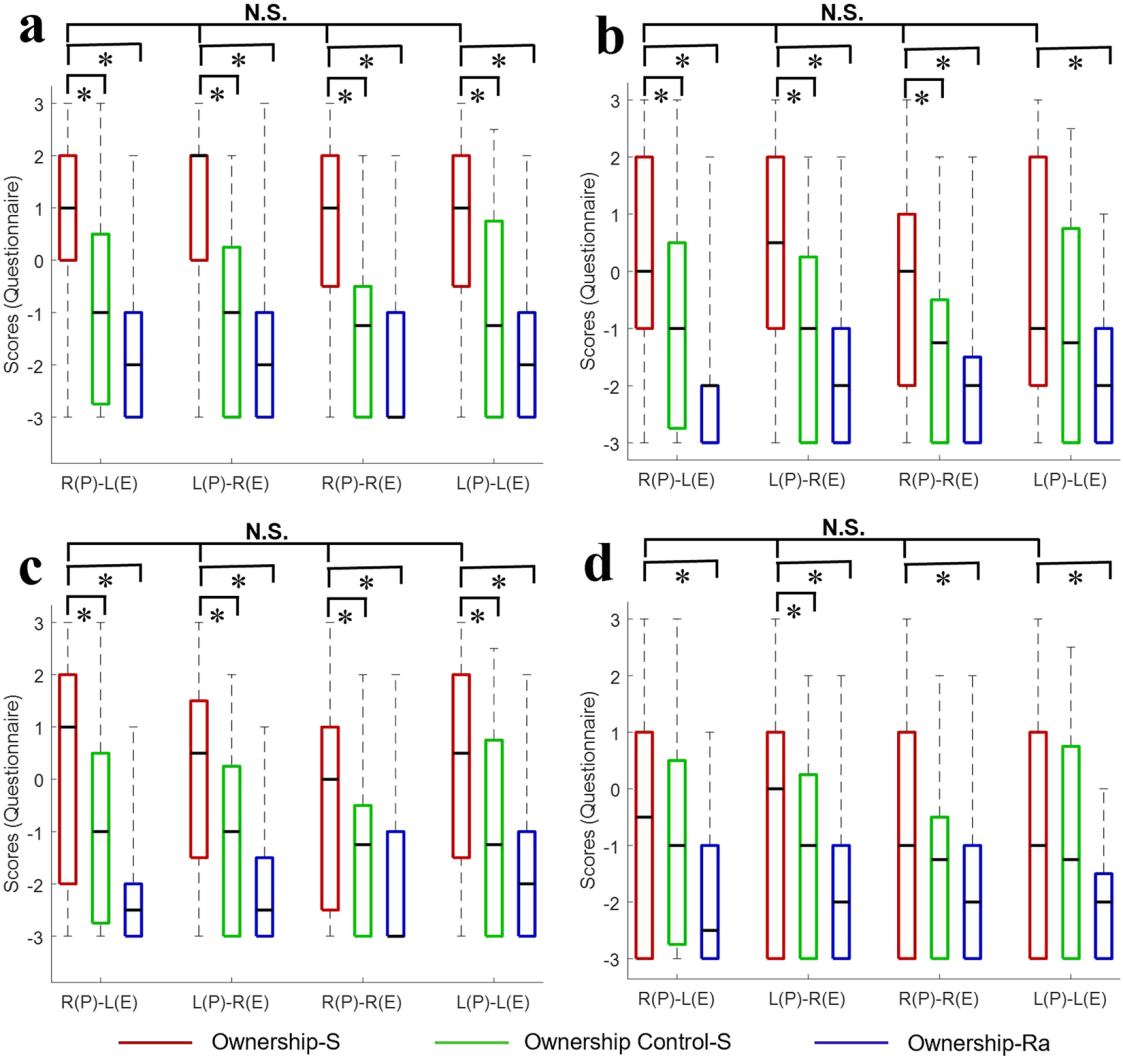
Ownership results for four questions in all of the conditions, showing the results of (**a**) Q_1_, (**b**) Q_3_, (**c**) Q_6_, and (**d**) Q_9_. The four experimental conditions are shown on the abscissa, and the ordinate shows the median scores in the question on the sense of ownership. Bold lines indicate the median; upper and lower limits of the box plot indicate the 75^th^ and 25^th^ percentiles, respectively. Error bars represent the whole range of the ratings of the statement. R: right hand. L: left hand. P: the participant. E: the experimenter. Ownership-S: ownership statement in the synchronous-movement condition. Ownership Control-S: ownership control statement in the synchronous-movement condition. Ownership-Ra: ownership statement in the random movement condition. N.S.: no significant difference. *p < 0.001.

The median and percentile (25%; 75%) of a perceived sense of ownership ratings are shown in Table 4 and Fig. 3. To examine the elicited sense of ownership, we compared the ownership statements with the corresponding control statements in the synchronous conditions and with the corresponding ratings in the random conditions with the Wilcoxon signed-rank test (Table 5 and Fig. 3). Then, we used a Bayesian Wilcoxon signed-ranks test^25^ to measure the differences in the ownership statements, including Q_1_, Q_3_, Q_6_, and Q_9_, between the mirrored and non-mirrored conditions. The Bayesian analysis used a Dirichlet process prior as a further test of the null results. The null hypothesis, H_0_, is that there is no significant difference in the ownership statement between mirrored and non-mirrored conditions.

**Table 4:** Median and percentiles [25%; 75%] for ownership ratings in all conditions.

Condition		Item				
		Q_1_	Q_3_	Q_6_	Q_9_	Q_C_
Synchronous condition	R(P)–L(E)	1[0;2]	0[–1;2]	1[–2;2]	–0.5[–3;1]	–1[–2.63;0.5]
	L(P)–R(E)	2[0;2]	0.5[–1;2]	0.5[–1.25;1.25]	0[–3;1]	–1[–3;0.13]
	R(P)–R(E)	1[–0.25;2]	0[–2;1]	0[–2.25;1]	–1[–3;1]	–1.5[–3;–0.5]
	L(P)–L(E)	1[–0.25;2]	–1[–2;2]	0.5[–1.25;2]	–1[–3;1]	–1.5[–3;0.63]
Random condition	R(P)–L(E)	–2[–3;–1]	–2[–3;–2]	–2.5[–3;–2]	–2.5[–3;–1]	–2[–3;–1]
	L(P)–R(E)	–2[–3;–1]	–2[–3;–1]	–2.5[–3;–1.75]	–2[–3;–1]	–2[–3;–0.88]
	R(P)–R(E)	–3[–3;–1]	–2[–3;–1.75]	–3[–3;–1]	–2[–3;–1]	–2[–1;–3]
	L(P)–L(E)	–2[–3;–1]	–2[–3;–1]	–2[–3;–1]	–2[–3;–1.75]	–2[–3;–1]

R: right hand. L: left hand. P: participant. E: experimenter. C: control statement.

**Table 5:** Comparisons between ownership ratings and ownership control ratings for synchronous conditions, and between ownership ratings of synchronous and random conditions.

Item	Condition							
	R(P)–L(E)		L(P)–R(E)		R(P)–R(E)		L(P)–L(E)	
	Z	*p*	Z	*p*	Z	*p*	Z	*p*
Q_1_ vs. Q_C_	4.462	<0.0001	4.752	<0.0001	4.313	<0.0001	4.252	<0.0001
Q_1-S_ vs. Q_1-Ra_	4.325	<0.0001	4.249	<0.0001	4.265	<0.0001	4.743	<0.0001
Q_3_ vs. Q_C_	2.653	0.007	4.997	<0.0001	4.015	<0.0001	2.222	0.025
Q_3-S_ vs. Q_3-Ra_	4.175	<0.0001	3.791	<0.0001	4.721	<0.0001	3.525	<0.001
Q_6_ vs. Q_C_	4.160	<0.0001	3.242	<0.001	3.277	<0.001	3.273	<0.001
Q_6-S_ vs. Q_6-Ra_	4.322	<0.0001	4.584	<0.0001	4.056	<0.0001	4.581	<0.0001
Q_9_ vs. Q_C_	1.823	0.069	3.037	0.002	2.304	0.0199	1.282	0.207
Q_9-S_ vs. Q_9-Ra_	3.744	<0.0001	3.110	0.001	3.153	0.001	4.343	<0.0001

R: right hand. L: left hand. P: participant. E: experimenter. C: control statement. S: synchronous condition. Ra: random condition.

Q_1_ and Q_6_ are ‘I felt as if I was looking at my own hand’ and ‘I felt as if the experimenter’s hand was my hand’. These questions are more directly related to an illusory feeling of ownership^3,33,34^. The medians of Q_1_ and three medians of Q_6_ in the synchronous conditions were positive (Table 4, Fig. 3a,c); only the median of Q_6_ in Right(P)–Right(E) was equal to 0, indicating a subjective perception of the sense of ownership^21^. The median scores of Q_1_ and three medians of Q_6_ in the synchronous conditions were also significantly higher than the medians for the corresponding control statements in synchronous conditions and the ownership ratings in the random conditions (all *p* < 0.001; Table 5). The questionnaire data clearly indicated that participants in the mirrored and non-mirrored movement conditions perceived a significant sense of ownership when the movement was synchronous (all *p* < 0.001; Table 5), but not when it was temporally random.

Q_3_ is ‘It seems as if I was sensing the movement of my finger in the location where the experimenter’s finger moved’, which concerns a sense of location^33,34^. The medians of Right(P)–Left(E), Left(P)–Right(E), and Right(P)–Right(E) were significantly higher than those of the corresponding control statements in the synchronous conditions and the ownership statements in the random conditions (all *p* < 0.001; Table 5 and Fig. 3b). The median score of Left(P)–Left(E) was higher than that of the corresponding control statements (*p* = 0.025) and significantly higher than that of the ownership statement in the random conditions (*p* < 0.001), although the median was −1.00.

Q_9_ is ‘I felt as if the experimenter’s hand was part of my body’, which differs from ‘I felt as if the rubber hand was part of my hand’ in the rubber hand illusion^4,5^. The medians of Q_9_ in Left(P)–Right(E) were significantly higher than those of the corresponding control statements in the synchronous conditions (*p* = 0.002) and the ownership statement in the random conditions (*p* = 0.001). The medians of Right(P)–Left(E), Right(P)–Right(E), and Left(P)–Left(E) were significantly higher than the corresponding ratings in the random conditions (*p* ≤ 0.001; Table 5 and Fig. 3d), and higher than those of the corresponding control statements (*p = *0.069, 0.0199, and 0.207).

To compare the overall statements regarding sense of ownership of mirrored and non-mirrored conditions, except for Q_9_, we used the mean value of responses to the ownership questions. Q_9_ was excluded because of its improper design, as described below. After Bayesian analysis, the Bayes factors of the P(H_0_|Data) for mirrored and non-mirrored conditions were 0.196 (Right(P)–Left(E) vs. Right(P)–Right(E)), 0.424 (Right(P)–Left(E) vs. Left(P)–Left(E)), 0.147 (Left(P)–Right(E) vs. Right(P)–Right(E)), and 0.352 (Left(P)–Right(E) vs. Right(P)–Right(E)). According to Jeffreys^32^, there is anecdotal evidence of similarity between the non-mirrored condition of Left(P)–Left(E) and the mirrored condition of Right(P)–Left(E) and Left(P)–Right(E) and moderate evidence of similarity between the non-mirrored condition of Right(P)–Right(E) and mirrored conditions of Right(P)–Left(E) and Left(P)–Right(E). Therefore, this result supports the null hypothesis H_0_, at least as a tendency. That is, the lack of a significant difference indicates that synchronous movements, rather than the mirrored movements of visual information, would elicit a sense of ownership during interpersonal synchronization.

### Correlation between agency and ownership

In addition, to analyse whether the senses of agency and ownership scores were correlated in all the synchronous conditions, we ran a correlation analysis in which we calculated the mean value of the agency and ownership statements, A Spearman’s rho test was used to test significance because of the non-parametric data^4,5^. The result showed close correlations between the agency and ownership statements in all synchronous conditions: (Right(P)–Left(E) condition, *r* = 0.294, *n* = 32, *p* = 0.05; Left(P)–Right(E) condition, *r* = 0.547, *n* = 32, *p* = 0.0005; Right(P)–Right(E) condition, *r* = 0.418, *n* = 32, *p* = 0.009; and Left(P)–Left(E) condition, *r* = 0.583, *n* = 32, *p* = 0.002).

## Discussion

The present study investigated whether the elicitation of agency and ownership during interpersonal synchronization is caused by the synchronous movements or mirror-symmetrical movements of another person. The absence of significant time-series differences indicated that the agency and ownership ratings in all the synchronous conditions, including the mirrored and non-mirrored conditions, were comparable. According to the questionnaire results, participants seemed to experience agency and ownership during interpersonal synchronization in both the mirrored and non-mirrored conditions. This indicates that interpersonal synchronization—including mirrored and non-mirrored movements—elicits the senses of agency and ownership. These results answer the remaining question of whether another person’s synchronous movements—rather than mirrored movements—are crucial for participants to experience these senses.

As measured by Q_2_ and Q_11_ in the present study, agency was related to feelings of being able to move the rubber hand and control it^35,36^. The positive median of Q_2_ and Q_11_ responses and their significant differences from those of the control statements and random statements showed elicitation of a sense of agency. Furthermore, the results of Q_5_ (relating to the sense of causing the viewed movement) also indicated that participants perceived a sense of agency. The positive median of Q_8_ scores and the significant differences from the control statements were consistent with those of Q_2_, Q_5_, and Q_11_, although the median was higher than, but not significantly different from, those of responses to the random statements. This may be expected even in random conditions because people synchronize their movements as soon as they exchange sensory information;^37,38^ it is easier to move synchronously together, and it also ‘makes us feel good about ourselves’^39,40^.

Furthermore, the lack of significant differences in responses to the agency statement between mirrored and non-mirrored conditions indicates that synchronous movements, rather than mirror-symmetrical movements, elicit a sense of agency. That is, the temporal synchronous perception during interpersonal synchronization could affect multisensory integration to induce this elicitation. Moreover, such processing would be related to the temporal integration window on which temporal synchronous perceptions depend. Some studies investigated the temporal integration window during the elicitation of agency and they found that a delay between an action and its feedback can generate the sense of agency^41,42^. The temporal integration window of perceived agency is even recalibrated along with perceived sensorimotor simultaneity during recalibrated training^11,12,14,15,43^, and sense of agency can in turn influence temporal recalibration^44,45^. However, some limitations should be noted, and these need to be resolved in our future work. According to the Bayesian analysis, the value of P(H_0_|Data) provides moderate support for the null hypothesis, H_0_, of similarity of responses in the mirrored condition of Left(P)–Right(E) and non-mirrored conditions of Right(P)–Right(E) and Left(P)–Left(E), whereas the value of P(H_0_|Data) only shows a tendency towards similarity between the mirrored condition of Right(P)–Left(E) and non-mirrored conditions of Right(P)–Right(E) and Left(P)–Left(E).

The elicitation of agency is consistent with that reported in previous work, including the study of person–person interactions by Zhou *et al*.^9^ and studies of human–rubber hand interactions^6–8^ (Table 1). In the human–rubber hand interactions, researchers have found that the synchronous movements of a rubber hand, placed congruently with a participant’s hand, elicited a sense of agency in the participant^4–8^, and that a sense of agency was elicited when the participant was interacting but viewing the synchronous movements of a 180° rotated rubber hand in mirror-and non-mirror-symmetrical ways, as if it was another person’s right or left hand^6–8^ (Table 1).

The consistency of the results from all of these studies suggests that a sense of agency is related to the temporal synchrony of movements between partners in an interaction (whether person–person or human–rubber), but not to mirrored movements *per se*. It is well known that a sense of agency is elicited when one is the agent of one’s own actions. Some studies have reported that the congruence of self-generated movements and perceptions of feedback from the temporal synchrony of movements play a role in eliciting a sense of agency^27,46^. In the synchronous conditions of the present study, participants observed a match between the hand movements they performed and those of the experimenter. The temporal synchrony in such interactions may imply a connection between the participant’s intention to move and the perceived movements of the experimenter^47^, and perhaps this connection elicits a sense of agency.

The questions on the sense of ownership (Q_1_ and Q_6_ in the present study) are usually used to measure the ownership illusion. The positive median of responses to these questions and their significant difference from those of the control and random statements indicated the elicitation of a sense of ownership. The medians of Q_3_ were slightly higher than or equal to 0 in the mirrored conditions. These medians indicate that participants might have been uncertain whether their hand was in the same position as that of the experimenter. This is because 0 means ‘uncertain’ on the seven-point Likert scale (−3 = totally disagree, 0 = uncertain, +3 = totally agree). This kind of uncertainty seemed consistent with participants’ sense of hand location in Zhou *et al*.^9^, in which the median of the shifted hand position (*i.e*., 3.32) was slightly below the uncertain level (*i.e*., 4).

In the present study, the medians of Q_9_ were almost all negative and did not differ significantly from those of the control statements or random statements. This might have been caused by the improper design of Q_9_ itself. Participants did not respond as expected to Q_9_ and Q_6_ because Q_9_ reminded them to consider where their hand was. In addition, the rubber hand and virtual hand^48^, which do not belong to anyone, are difficult to think of as part of someone’s body, while another person’s hand might easily be considered a part of someone’s body. Therefore, we suggest that the questionnaire results indicate that participants experienced a sense of ownership.

Furthermore, the lack of significant difference in the ownership statements between the mirrored and non-mirrored conditions indicates that human synchronous movements, rather than human mirror-symmetrical movements, would elicit a sense of ownership. As for similar results in the elicitation of agency in the present study, we also suggest that such processing is related to the temporal integration window on which temporal synchronous perception depends. Some previous studies have reported that the senses of agency and ownership are temporally plastic^49–52^. However, some limitations should be noted that need to be resolved in our future work. According to the Bayesian analysis, the value of P(H_0_|Data) provides moderate support for the null hypothesis, H_0_, for the non-mirrored condition of Right(P)–Right(E) and mirrored conditions of Right(P)–Left(E) and Left(P)–Right(E), whereas the value of P(H_0_|Data) only shows a tendency in the non-mirrored condition of Left(P)–Left(E) and mirrored conditions of Right(P)–Left(E) and Left(P)–Right(E). Finally, no studies to date have investigated the relationship between the time delay in interpersonal synchronization and the elicitation of the senses of agency and ownership.

The result of sense of ownership is consistent with the human–human results of Zhou *et al*.^9^ but inconsistent with those of research on human–rubber hand interactions^6–8^ (Table 1). In the human–rubber hand interactions, researchers have found that the synchronous movements of a rubber hand, placed congruently with a participant’s hand, elicited a sense of ownership in the participant^4–8^, but not when the participant was interacting while viewing the mirror- and non-mirror-symmetrical synchronous movements of a 180° rotated rubber hand^6–8^ (Table 1). Thus, the experience of ownership found in the present study and by Zhou *et al*.^9^ as well as the lack of ownership experienced in human–rubber hand studies^6–8^ indicate that the temporal synchrony of movements is insufficient to explain the elicitation of ownership. Thus, synchronization with a human regardless of the mirrored and non-mirrored movements plays some role in the sense of ownership, but synchronization with a rubber hand does not. This suggests that a sense of ownership may play a role in human social functions rather than simply referring to the feeling that ‘my body belongs to me’^52^. Based on work with the rubber hand illusion and the virtual hand illusion, a sense of ownership may involve quite plastic, top-down processing when either the rubber hand or virtual hand is placed in a realistic position in relation to the observer/participant^30^. We speculate that a sense of ownership is part of the processing that occurs in social interactions and in the flexible top-down processing in face-to-face interactions. Such top-down processing may have caused the mean ratings for ownership to be lower than those for agency in the present study because participants used face-to-face interaction with the experimenter as well as the experimenter’s hand movements to make their final decisions of the feeling of ownership. In addition, it is necessary to discover the mechanism underlying the elicitation of the sense of ownership in interpersonal interactions. This mechanism remains unclear because it was not elicited when the participant was interacting while viewing the mirror- and non-mirror-symmetrical synchronous movements of a 180° rotated rubber hand^6–8^ (Table 1).

The correlation results show a close correlation between the agency and ownership statements in all synchronous conditions. The close correlation is consistent with the results of some recent studies^53–55^, which have suggested that the senses of agency and ownership could partly overlap at the neurofunctional level and have even proposed an ‘interactive’ model for the two senses, as the sense of ownership *per se* can act on the sense of agency attribution^55^.

In the present study, we investigated whether it is the human synchronous movements or human mirrored movements that made participants feel both a sense of agency and ownership. We compared the senses of agency and ownership in the mirrored synchronous and random conditions and in the non-mirrored synchronous and random conditions. To ensure comparability across the synchronous conditions, we established the consistency of synchronous hand movements between the participant and experimenter by tracking their hand movements. The results from the agency and ownership analyses indicated that it was synchronous movements regardless of mirroring that elicited senses of agency and ownership. Hence, the results also suggest that people could experience these senses not only from their own movements but also from others’ synchronous movements.

## Methods

### Participants

Computation of the sample size was performed with G-Power 3 (Heinrich Heine University—Institut für experimentelle psychologie; www.psycho.uni- duesseldorf.de/abteilungen/aap/gpower3). With respect to the perception of sense of ownership during interpersonal synchronization, we based our sample size estimation on a previous study^9^. As indicated by Zhou *et al*., the effect of interpersonal synchronization on the elicitation of sense of ownership has a Cohens’ d = 1.18 with a mean of 4.00 and standard deviation of 1.81 (modified for within-subject design). Assuming an anticipated effect size equal to 1.18, an α error probability of 0.05 and a power (1 – β error probability) of 0.95, the resulting total sample size is n = 12. Thus, based on this power analysis, we conservatively estimated a larger sample size and recruited 32 participants for the study. These 32 participants (15 females, 17 males; mean age: 23.9 years; range: 22–30 years) completed the experiment and were compensated for their participation. All participants were right-handed^23^ and none exhibited any difficulty moving their hands or fingers. All participants had normal or corrected-to-normal vision and no history of neurological disease. They were naive as to the experiment’s purpose. We obtained written informed consent from each participant prior to participation. The study was approved by the Ethics Committee of the Tokyo Institute of Technology and the methods were conducted in accordance with its approved guidelines.

### Design

This experiment assessed whether Synchrony and Movement type (mirrored vs. non-mirrored) influence the senses of agency and ownership during interpersonal synchronization. Synchrony between a participant’s and an experimenter’s hand movements was manipulated so that movements were either temporally congruent (*i.e*., the experimenter synchronously imitated the participant’s hand movement) or incongruent (*i.e*., temporally random). We manipulated movement type by seating the participant and experimenter opposite each other and having the experimenter move either the opposite (mirrored condition) or the same (non-mirrored condition) hand as the participant. We also had the participants move either their left and right hands; thus, the experiment had a 2 Synchrony (synchronous vs. random) × 2 Movement type (mirrored vs. non-mirrored) × 2 Participant’s hand (left vs. right) design. A fully factorial combination of these three factors produced eight conditions (Table 6). Participants followed these within-subject conditions in a random order.

**Table 6:** Summary of the experimental design and conditions.

Condition	Synchronous		Random	
Mirrored	R(P)–L(E)	L(P)–R(E)	R(P)–L(E)	L(P)–R(E)
Non-mirrored	R(P)–R(E)	L(P)–L(E)	R(P)–R(E)	L(P)–L(E)

R: right hand. L: left hand. P: the participant. E: the experimenter.

### Apparatus and procedure

**Figure 4:**
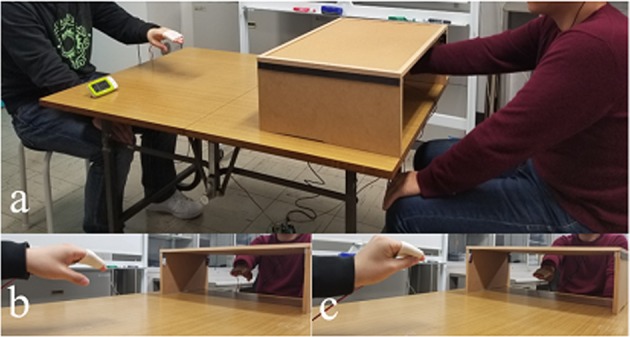
The experimental setting showing: (**a**) a participant and an experimenter, (**b**) a view of the wooden box, a participant’s hand, and the graph paper, (**c**,**d**) an illustration of the experimental task with the participant and the experimenter making (**c**) the open-hand motion and (**d**) the close-hand motion.

The experiment was conducted in a quiet experimental room at the Tokyo Institute of Technology. The participant sat comfortably at a table and put his/her hand into a wooden box with the dimensions 21 cm (h) × 40 cm (w) × 60 cm (d) (Fig. 4a). The box was placed on a table directly in front of the participant, in alignment with the sagittal body midline. Each participant was paired with an experimenter of the same sex, who sat on the opposite side of the rectangular table (75 cm × 120 cm) (Fig. 4a). The distance between the participant’s and experimenter’s fingertips was around 50 cm. We used a Count Down Digital Timer (TD-394; Tanita Co., Tokyo, Japan) and sensors (FSR402; Interlink Electronics Co., Camarillo, CA, USA) to track inverse changes in resistance in response to increases/decreases in applied force in relation to the time-series data during synchronous movements of the participant and experimenter.

Prior to the experiment, each participant was asked to read instructions on the procedure. The participant’s task was to open and close his or her hand (Fig. 4b,c) at approximately 1 s intervals. The participants received brief training in how to perform the appropriate open-and-close motion and how to use the digital timer for pacing. The timer counted down from 1 to zero minutes during training, but it was not used during experimental trials. During the experiment, sensors were used to track the timing of the participant’s and experimenter’s open-and-close hand movements, and the participant was asked to look at the experimenter’s hand movements. The order of conditions was counterbalanced among participants. The participants had a 2–3-minute break after each condition to prevent the previous experimental condition from influencing the next one. The experiment took approximately 90 minutes to complete.

In the synchronous mirrored condition, the participant was asked to perform the open-and-close motion with his/her right or left hand at approximately 1 s intervals for 60 s, and stare at the experimenter’s hand motions while keeping his/her own rhythm. The experimenter sat opposite the participant and synchronously moved his/her opposite hand in imitation of the participant’s hand movements. White noise helped the participant to focus on the hand movement task. On completion of the experiment, participants completed the 12-item questionnaire (Table 7).

**Table 7:** Questionnaire for agency and ownership.

Category	Statement	Order of questions
Agency Judgement	The experimenter’s hand moved just like I wanted it to, as if it was obeying my will.	2
	I felt as if I was causing the movement that I saw.	5
	Whenever I moved my finger, I expected the experimenter’s finger to move in the same way.	8
	I felt as if I was controlling the movements of the experimenter’s hand.	11
Ownership judgement	I felt as if I was looking at my own hand.	1
	It seems as if I was sensing the movement of my finger in the location where the experimenter’s finger moved.	3
	I felt as if the experimenter’s hand was my hand.	6
	I felt as if the experimenter’s hand was part of my body.	9
Agency Control	I felt as if the experimenter’s hand was controlling my will.	4
	I felt as if the experimenter’s hand was controlling the movement of my hand.	12
Ownership Control	I felt as if I no longer had a right/left hand, as if my right/left hand had disappeared.	7
	It appeared as if the experimenter’s hand was drifting towards my real hand.	10

In the random mirrored condition, the experimenter performed the hand movements in synchronization with temporally random sounds rather than in synchronization with the participant’s hand movements. Intervals between these sounds were randomly set between 0.9 and 1.5 s because participants’ intervals were approximately 1 s. The other procedures for this condition were the same as in the synchronous mirrored condition.

In the synchronous non-mirrored condition, the participants performed the open-and-close motion with their right or left hand, and the experimenter synchronously performed the motion with the same hand (right or left). The other procedures were the same as in the synchronous mirrored condition.

In the random non-mirrored condition, the experimenter performed the hand movements in synchronization with temporally random sounds rather than with the participant’s hand movements. The other procedures were the same as in the synchronous non-mirrored condition.

### Measures of agency and ownership

To assess the subjective experiences of agency and ownership, we used a 12-item questionnaire adopted from Braun *et al*.^30^, and Kalckert and Ehrsson^4^ and used in traditional rubber hand illusion experiments^3,56^ (Table 7). The questions were presented in a pseudo-randomized order and rated on a seven-point Likert scale (−3 = totally disagree, 0 = uncertain, +3 = totally agree). A Likert scale (printed on A4 paper) accompanying the verbal presentation of each statement was used to facilitate responses when necessary. The responses to four items were used to obtain a single value for the perceived senses of agency and ownership. The remaining four items were control statements, with two for agency and two for ownership^30^. Hence, if a sense of agency is induced, participants should give high scores on the sense of agency questions highly in the four synchronous conditions and lower scores for the agency control questions, as responses to these questions should not specifically be affected by the manipulation of agency. Similarly, high ownership questions and low or negative ownership control questions mean that a sense of ownership is induced.

### Data analysis

Prior to conducting data analyses, we used the Shapiro–Wilk test (*p* > 0.05) to see whether the data were normally distributed. Because several datasets failed to meet the criteria for normal distribution, we used appropriate non-parametric tests. We used the Wilcoxon signed-rank test for pairwise comparisons. All tests were two-tailed, and all analyses were conducted using the R software package (R Studio 1.1.419, Inc., Boston, MA, USA).

We calculated time-series data for each participant’s and experimenter’s synchronous and random hand movements to ensure there was no difference between their movements in synchronous conditions. Then, we calculated participants’ perceived senses of agency and ownership during synchronous movements with the experimenter, as follows. First, we compared agency and ownership scores with their respective control statements for each experimental condition to see whether there was a significant sense of agency or ownership in each synchronous condition. Second, we compared agency and ownership scores from synchronous conditions with those from random conditions to test for significant differences between synchronous and random conditions. Third, we examined whether the experimenter’s mirrored movements were needed to elicit a sense of agency or ownership during interpersonal synchronization, by using a Bayesian version of the Wilcoxon signed-ranks test^25^. Finally, we measured the correlation of the agency and ownership statements in all the synchronous conditions by a Spearman rho test because of the non-parametric data^4,5^.

## Data Availability

The datasets generated and/or analysed during the current study are available from the corresponding author on reasonable request.
